# Validation of the vault prediction model based on the sulcus-to-sulcus diameter and lens thickness: a 925-eye prospective study

**DOI:** 10.1186/s12886-022-02698-z

**Published:** 2022-11-30

**Authors:** Qiu-Jian Zhu, Xiao-Ying Xing, Man-Hui Zhu, Lie Ma, You Yuan, E. Song

**Affiliations:** 1 Department of Ophthalmology, The Second Affiliated Hospital of Soochow University, Suzhou, China; 2 Department of Ophthalmology, Lixiang Eye Hospital of Soochow University, Suzhou, China; 3 Suzhou Eye Hospital, Suzhou, China

**Keywords:** ICL implantation, Prediction formula, Vault, Sulcus to sulcus, UBM

## Abstract

**Background:**

To verify the accuracy and stability of the prediction formula based on the ciliary sulcus diameter and lens thickness and to analyse factors influencing the prediction results.

**Methods:**

In total, 925 eyes from 506 subjects were enrolled in this prospective study between July 1, 2020, and June 30, 2021. Subjects were divided into four seasons, each spanning three months. The target vault was set to be between 300 μm and 700 μm according the prediction formula. The actual vault was measured one month postoperatively. The Bland–Altman test, 95% confidence intervals (95% CI) and 95% limits of agreement (95% LoA) were used to evaluate the agreement between the predicted vault and the actual vault. Eyes with absolute prediction errors greater than 300 μm were further analysed.

**Results:**

The mean predicted vaults for the four seasons were 503 ± 99, 494 ± 96, 481 ± 92 and 502 ± 93 μm, while the mean actual vaults were 531 ± 189, 491 ± 179, 464 ± 179 and 529 ± 162 μm, respectively. The predicted and actual vaults of the overall subjects were 493 ± 95 and 500 ± 180 μm, respectively. Of the 925 eyes, 861 eyes (93.08%), 42 eyes (4.54%), and 22 eyes (2.38%) showed a normal vault, high vault, and low vault, respectively. Bland–Altman plots showed that the mean difference between the actual vault and predicted vault overall (± 95% LoA) was 6.43 ± 176.2 μm (-339 to 352 μm). Three UBM features may lead to large prediction errors (more than 300 μm): wide iris-ciliary angle (ICA), iris concavity and anteriorly positioned ciliary body.

**Conclusions:**

This study demonstrated the accuracy and stability of the prediction formula through the validation of a large sample size and a long time span. Wide ICA, iris concavity and anteriorly positioned ciliary body may have an effect on vault.

## Background

Uncorrected myopia is the leading cause of visual impairment worldwide, and the incidence of myopia is increasing [1, 2]. It is expected that there will be approximately 4.758 billion myopic people and 938 million highly myopic people worldwide by 2050 [2]. Corneal refractive surgery is currently the mainstream myopia correction procedure, but it is limited by corneal thickness and is not suitable for high myopia, thin corneas and abnormal corneal morphology [3].

EVO implantable collamer lens (ICL; STAAR Surgical Co, Monrovia, California) implantation is an intraocular refractive surgery and has become the preferred surgical correction strategy for high myopia and corneal abnormalities due to the lack of corneal excision [4]. Many studies have proven that ICL implantation has good stability, safety and clinical efficacy [5–9]. However, various complications have still been reported from time to time, and most of them were related to improper vault. For example, acute angle-closure glaucoma, iris atrophy and pigment dispersion syndrome are often associated with excessive vault, whereas insufficient vault might cause anterior subcapsular cataract and ICL rotation [10–16].

There are four sizes of ICLs (12.1 mm, 12.6 mm, 13.2 mm and 13.7 mm), and selection of the right size is the key to obtaining the ideal vault. However, how to select is still controversial. The manufacturer's recommended selection strategy is based on white to white (WTW) and anterior chamber depth (ACD), which is also recommended by the FDA (Visian ICL Product Information: Visian ICL For Myopia. Available at http://www.accessdata.fda.gov/cdrh_docs/pdf3/p030016c.pdf). However, some studies suggest that the accuracy of this method is not satisfactory [17–19]. In recent years, anterior segment optical coherence tomography (ASOCT) has been widely used in the measurement of anterior segment parameters and ICL size selection due to its high precision and repeatability [19–21]. However, ASOCT is an additional examination, and since it cannot detect the ciliary sulcus, this examination has no guiding effect for surgery other than vault. At present, ultrasound biomicroscopy (UBM) is the only device that can detect ciliary sulcus. As ciliary body lesions need to be excluded, UBM is a necessary examination before ICL. Moreover, since ICL is placed in the ciliary sulcus, which can be directly detected by UBM, UBM might be more suitable for predicting vault.

Recently, we proposed a vault prediction method based on the sulcus-to-sulcus diameter and LT [22]. In the present study, we verified the accuracy and effectiveness of this method in vault prediction during ICL implantation by using a large sample size and long-term observation.

## Methods

### Study design and participants

This prospective study was approved by the Lixiang Eye Hospital of Soochow University Institutional Review Board and adhered to the tenets of the Declaration of Helsinki. Informed consent was obtained from every subject before surgery. All subjects were recruited from the refractive surgery centre of Lixiang Eye Hospital between July 1, 2020, and Jane 30, 2021. A total of 925 eyes from 506 subjects were enrolled in this study. With three months as a season, we divided this study into four stages: from July 1, 2020, to September 30, 2020, for the first season; from October 1, 2020, to December 31, 2020, for the second season; from January 1, 2021, to March 31, 2021, for the third season; and from April 1, 2021, to June 30, 2021, for the fourth season. Each season was analysed separately.

The inclusion criteria for this study included 18 years ≤ age ≤ 45 years; myopia between -0.50 and -18.00 DS and astigmatism between 0 and -6.00 DC; stable refraction; endothelial cell density ≥ 2000 cells/mm^2^; anterior chamber depth ≥ 2.8 mm. Exclusion criteria were eyes with diseases such as keratoconus, endothelial corneal dystrophy, severe dry eye, infection, glaucoma, cataract; fundal diseases that significantly affect vision, or severe psychological diseases.

### Examination and surgery

All subjects underwent a complete preoperative examination, including comprehensive optometry, visual acuity, slit lamp examination, intraocular pressure, dilated fundus examination by a three-mirror lens, Pentacam (Oculus, Germany), IOLMaster 700 (Carl Zeiss Meditec AG, Jena, Germany), OPD-Scan III (Nidek Technologies, Gamagori, Japan) and ultrasound biomicroscopy (UBM; SW-3200 L; SUOER, Tianjin, China), as described previously [22]. The vault measured by Pentacam one month after the operation was adopted for analysis. Low vault, normal vault and high vault were defined as 0 to 199 μm, 200 to 800 μm and 801 μm or more, respectively. Lens thickness (LT) was measured automatically by an IOLMaster 700.

The horizontal and vertical sulcus-to-sulcus (STS) diameters were measured using the UBM equipped with a 50 MHz transducer. During measurement, subjects were asked to laterally fixate their eyes on a 4 m high ceiling target to avoid accommodation. Cross-sectional images were obtained in the following two directions: vertical (up-down, 90°) and horizontal (temporal-nasal 0°). Finally, images with the largest sulcus-to-sulcus diameters were used for analysis.

All surgeries were performed using standard surgical procedures and were conducted by the same experienced physician (YY). In brief, a temporal 2.8-mm corneal incision was made after topical anaesthesia (proxymetacaine hydrochloride, Nanjing, China). A V4c model ICL (STAAR Surgical Co, Monrovia, California) was inserted into the anterior chamber following viscoelastic-agent (hyaluronic acid, Shanghai, China) injection. Then, the ICL was placed in the posterior chamber and adjusted to the planned position. Next, the viscoelastic agent was completely removed from the eye using a manual I/A instrument. All surgeries were uneventful, and no intraoperative complications were observed. Following surgery, tobramycin 0.3% dexamethasone 0.1% eye drops (Tobradex, Alcon, USA) were administered four times daily for the first 5 days; dosages were decreased every 5 days.

Finally, eyes with absolute prediction errors greater than 300 μm were further analysed.

### Lens selection

In this study, the ICL sizes were selected based on the previously published prediction formula: central vault (μm) = -1369.05 + 657.121 × ICL size- 287.408 × horizontal STS—432.497 × crystalline LT—137.33 × vertical STS, and the target vault was set to be between 300 μm and 700 μm [22]. The power calculations for the ICL were performed according to the manufacturer’s guidelines using a modified vertex formula [23].

### Statistical analysis

Data analysis was conducted by SPSS 18.0 (IBM Corp., New York, NY, USA). The data are expressed as the means ± standard deviations (SDs) since the Kolmogorov–Smirnov test was performed for all measurement data and all parameters had a normal distribution. The Bland–Altman test, 95% confidence intervals (95% CI) and 95% limits of agreement (95% LoA) were used to evaluate the agreement between the predicted vault and the actual vault. All tests were 2-tailed, and a p value of less than 0.05 was considered significant.

## Results

In this study, a total of 925 eyes from 506 subjects were enrolled, including 221 eyes of 119 subjects in the first season, 200 eyes of 112 subjects in the second season, 302 eyes of 166 subjects in the third season, and 202 eyes of 109 subjects in the fourth season. Table 1 shows the baseline characteristics of the study population.

**Table 1:** Baseline clinical characteristics of the study eyes. Mean ± SD

Characteristics	Season 1	Season 2	Season 3	Season 4	Overall
Number, subjects/eyes	119/221	112/200	166/302	109/202	506/925
Age, years	26.60 ± 6.40	27.96 ± 5.62	27.73 ± 5.74	25.73 ± 6.77	27.07 ± 6.17
Sex (male/female)	46/73	51/61	68/98	39/70	204/302
Refractive errors (D)					
Spherical	-7.15 ± 2.57	-7.37 ± 2.50	-7.16 ± 2.43	-7.60 ± 2.35	-7.30 ± 2.46
Cylindrical	-1.25 ± 0.96	-1.40 ± 0.83	-1.22 ± 0.98	-1.37 ± 0.96	-1.30 ± 0.94
Spherical equivalent	-7.78 ± 2.54	-8.07 ± 2.53	-7.78 ± 2.46	-8.29 ± 2.51	-7.95 ± 2.51
Keratometric value (D)					
Flat K	42.86 ± 1.24	43.07 ± 1.34	42.93 ± 1.46	42.86 ± 1.33	42.93 ± 1.36
Steep K	44.41 ± 1.51	44.59 ± 1.44	44.40 ± 1.58	44.49 ± 1.49	44.46 ± 1.51
Mean K	43.63 ± 1.31	43.83 ± 1.35	43.66 ± 1.46	43.67 ± 1.36	43.69 ± 1.38
STS diameter (mm)					
Vertical	12.09 ± 0.43	11.93 ± 0.40	11.88 ± 0.42	11.97 ± 0.45	11.96 ± 0.43
Horizontal	11.64 ± 0.40	11.49 ± 0.41	11.48 ± 0.38	11.59 ± 0.42	11.54 ± 0.41
IOP (mm Hg)	13.25 ± 2.41	13.22 ± 2.68	13.53 ± 2.73	13.42 ± 2.64	13.37 ± 2.62
AL (mm)	26.77 ± 1.38	26.64 ± 1.26	26.61 ± 1.31	26.91 ± 1.21	26.72 ± 1.30
ACD (mm)	3.26 ± 0.20	3.24 ± 0.22	3.22 ± 0.24	3.27 ± 0.24	3.24 ± 0.23
WTW diameter (mm)	11.69 ± 0.33	11.56 ± 0.38	11.56 ± 0.41	11.65 ± 0.38	11.61 ± 0.38
ICL size (12.1/12.6/13.2/13.7)	8/103/107/3	15/110/74/1	19/201/81/1	6/128/66/2	48/542/328/7
Crystalline LT (mm)	3.68 ± 0.24	3.70 ± 0.22	3.67 ± 0.23	3.61 ± 0.22	3.67 ± 0.23

*STS* Sulcus to sulcus, *IOP* Intraocular pressure, *AL* Axial length, *ACD* Anterior chamber depth, *WTW* White to white, *LT* Lens thickness

### Vault values

The predicted and actual vault values in the four seasons are shown in Table 2. The mean predicted vaults for the four seasons were 503 ± 99, 494 ± 96, 481 ± 92 and 502 ± 93 μm, while the mean actual vaults were 531 ± 189, 491 ± 179, 464 ± 179 and 529 ± 162 μm, respectively. The predicted and actual vaults of the overall subjects were 493 ± 95 and 500 ± 180 μm, respectively.

**Table 2:** Predicted and actual vaults in the four seasons. Mean ± SD (range)

	Season 1	Season 2	Season 3	Season 4	Overall
Predicted vault, μm	503 ± 99 (308, 700)	494 ± 96 (301, 694)	481 ± 92 (302, 696)	502 ± 93 (306, 694)	493 ± 95 (301, 700)
Actual vault, μm	531 ± 189 (50, 1150)	491 ± 179 (0, 920)	464 ± 179 (0, 1060)	529 ± 162 (200, 990)	500 ± 180 (0, 1150)
Number, eyes	221	200	302	202	925

### Prediction accuracy

**Fig. 1:**
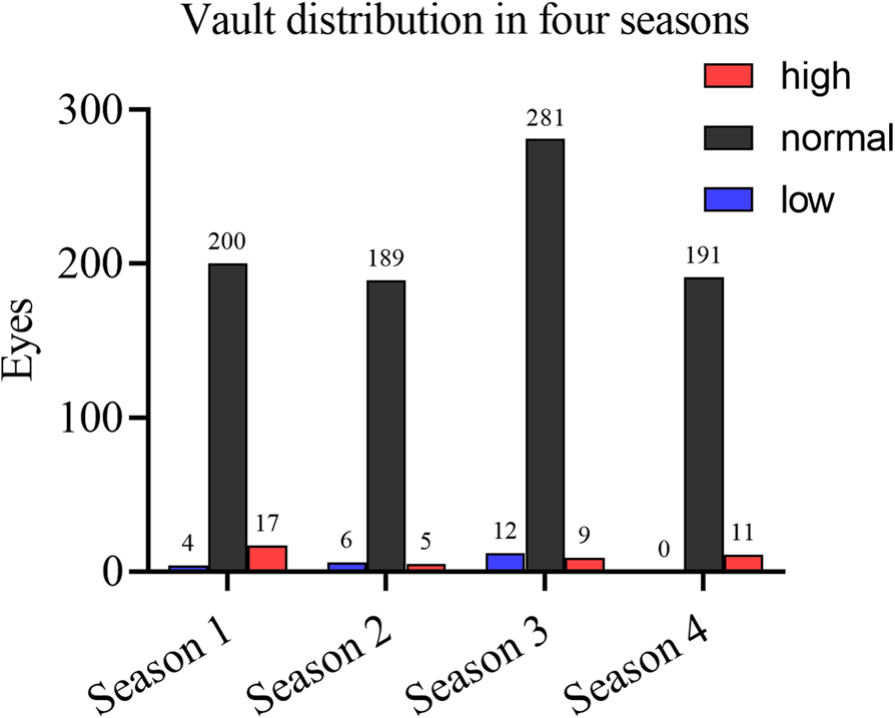
Actual vault distribution in four seasons. High vault, more than 800 μm; normal vault, 200 to 800 μm; low vault, less than 200 μm

Table 3 and Fig. 1 show the actual distribution of normal, high and low vaults in the four seasons. Of the 925 eyes, 861 eyes (93.08%), 42 eyes (4.54%), and 22 eyes (2.38%) showed a normal vault, high vault, and low vault, respectively. This distribution was 200 eyes (90.50%), 17 eyes (7.69%), and 4 eyes (1.81%) in the first season; 189 eyes (94.50%), 5 eyes (2.5%), and 6 eyes (3.00%) in the second season; 281 eyes (93.05%), 9 eyes (2.98%), and 12 eyes (3.97%) in the third season; and 191 eyes (94.55%), 11 eyes (5.45%), and 0 eyes (0%) in the fourth season.

**Table 3:** Distribution of the different types of vault in the four seasons. Number (percentage)

	Season 1	Season 2	Season 3	Season 4	Overall
Low	4 (1.81%)	6 (3.00%)	12 (3.97%)	0 (0.00%)	22 (2.38%)
Normal	200 (90.50%)	189 (94.50%)	281 (93.05%)	191 (94.55%)	861 (93.08%)
High	17 (7.69%)	5 (2.50%)	9 (2.98%)	11 (5.45%)	42 (4.54%)

### Bland–Altman analysis

**Fig. 2:**
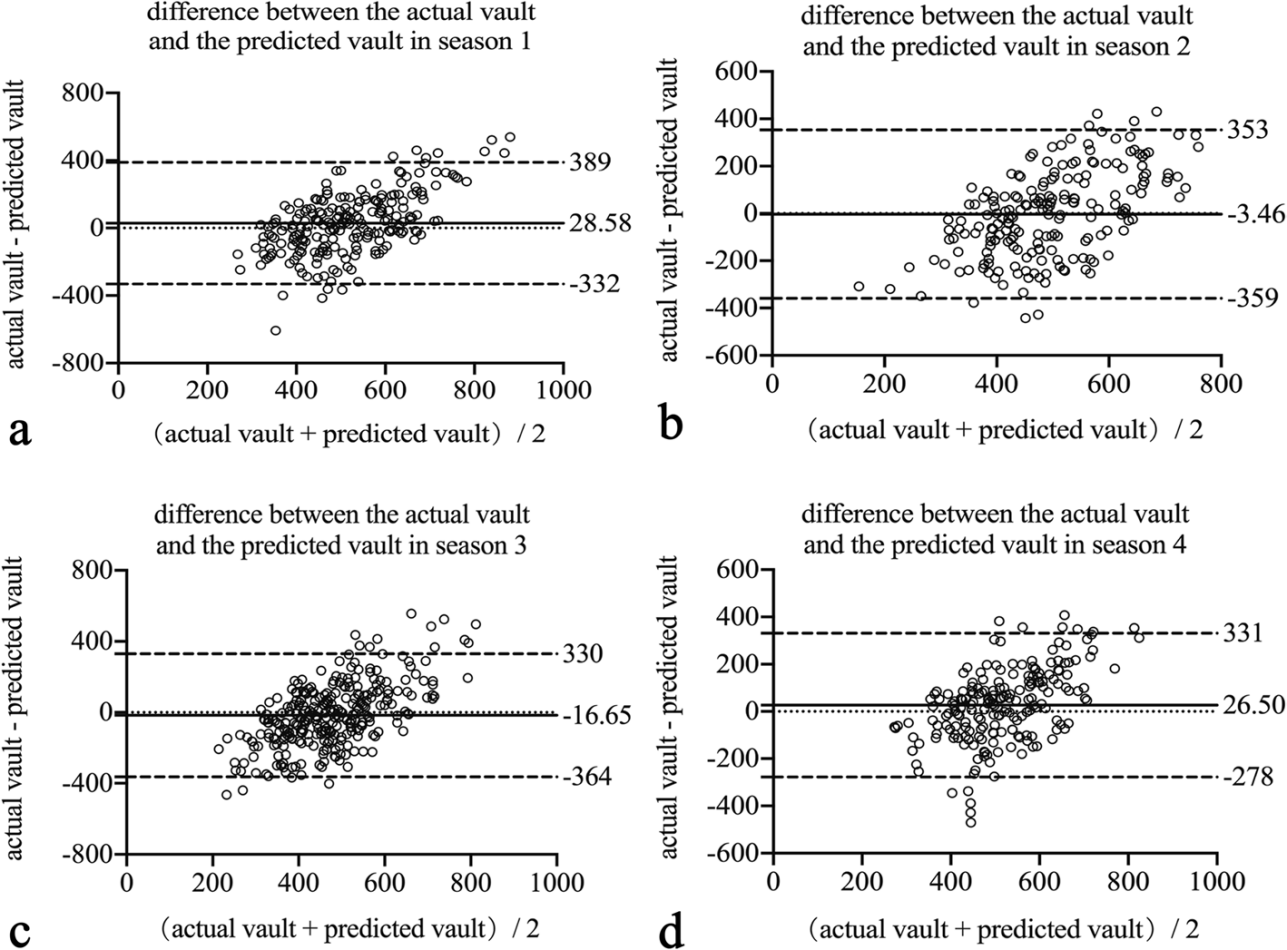
Bland–Altman plots showing the difference between the actual and predicted implantable collamer lens (ICL) vault divided by the mean of the actual and predicted ICL vault in four seasons. The dotted lines represent mean differences between the achieved and predicted implantable collamer lens (ICL) vault; dashed lines are the upper and lower borders of the 95% limits of agreement (mean difference ± 1.96 multiplied by the standard deviation of the mean difference). a, b, c and d represent the four seasons

Figure 2 and Table 4 show the agreement and difference between the actual vault and predicted vault in the four seasons. Bland–Altman plots show that the mean difference between the actual vault and predicted vault in the four seasons (± 95% LoA) was 28.58 ± 184.03 μm (-332 to 389 μm), -3.45 ± 181.61 μm (-359 to 353 μm), -16.65 ± 177.09 μm (-364 to 330 μm), and 26.50 ± 155.53 μm (-278 to 331 μm).

**Table 4:** Difference between the actual vault and the predicted vault in the four seasons. μm

	Season 1	Season 2	Season 3	Season 4	Overall
Difference	28.58	-3.45	-16.65	26.50	6.43
SD	184.03	181.61	177.09	155.53	176.20
Range	-607, 539	-443, 430	-465, 556	-471, 407	-607, 556
25%, 75%	-92, 152	-134, 131	-127, 98	-77, 123	-110, 115
95% CI	52.98, 4.19	-28.77, 21.87	-36.70, 3.40	4.92, 48.08	-4.93, 17.80
95% LoA	-332, 389	-359, 353	-364, 330	-278, 331	-339, 352

*SD* Standard deviation, *CI* Confidence interval, *LoA* Limit of agreement

### UBM features

There were 36 eyes with an actual vault more than 300 μm lower than the predicted vault (overestimated group) and 54 cases with an actual vault more than 300 μm higher than the predicted vault (underestimate group). Table 5 summarizes four UBM features that may lead to large prediction errors (more than 300 μm): wide iris-ciliary angle (ICA), iris concavity, anteriorly positioned ciliary body and ciliary body cyst.

**Table 5:** Characteristics of subjects with absolute difference between actual vault and predicted vault greater than 300 μm. Number (percentage)

Characteristics	Actual vault—predicted vault < -300 μm, *N* = 36	Actual vault—predicted vault > 300 μm, *N* = 54	*P* value
Wide iris-ciliary angle (ICA) ^a^	26 (72.22%)	2 (3.70%)	< 0.01
Iris concavity ^b^	7 (19.44%)	0 (0%)	< 0.01
Anteriorly positioned ciliary body ^c^	0 (0%)	28 (51.85%)	< 0.01
Ciliary body cyst ^d^	0 (0%)	6 (11.11%)	0.077

Fisher exact probability chi-square test

**Fig. 3:**
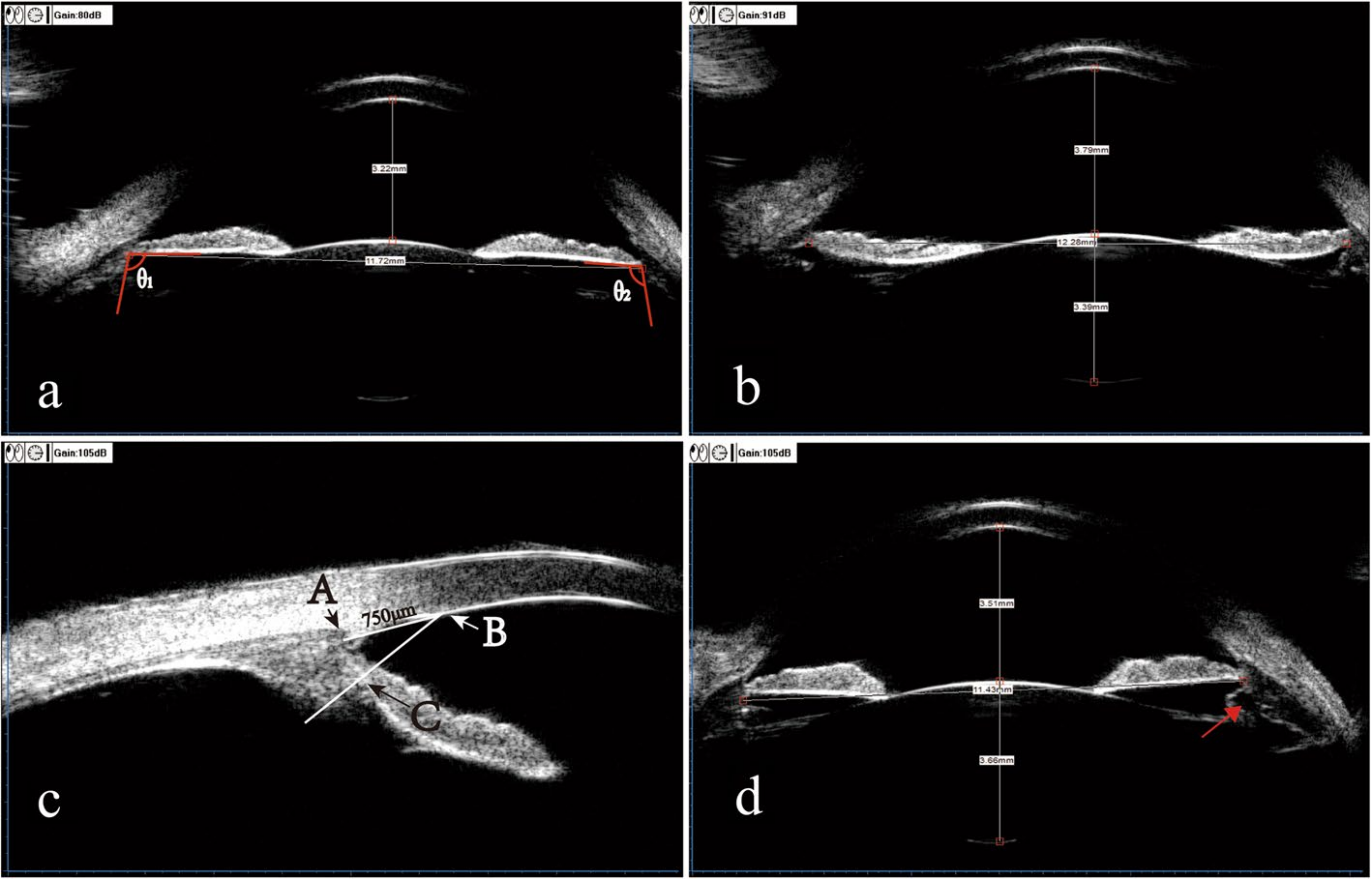
Four UBM features that may affect implantable collamer lens (ICL) vault: a wide iris-ciliary angle (ICA); angles θ1 and θ2 between the posterior iris surface and the anterior surface of the ciliary body measuring greater than 90°; **b** iris concavity, in which the iris bows backwards; **c** anteriorly positioned ciliary body. (B) is a point of the corneal endothelium 750 μm from the scleral spur (A), a line is drawn from (B) perpendicular to the posterior surface of the iris, intersects with the points (C), between (C) and the iris root, the ciliary body and iris contact, and the ciliary sulcus disappears; d ciliary body cyst, the red arrow points to the ciliary body cyst

^a^Refers to an angle between the iris and ciliary body greater than 90°. As described in Panel A of Fig. 3

^b^The iris is bowing backwards, as described in Panel B of Fig. 3

^c^The definition of an anteriorly positioned ciliary body was exhibited in at least two quadrants of the ciliary body, as described in Panel C of Fig. 3

^d^Ciliary body cyst was showed in Panel D of Fig. 3

### Secondary surgeries/adverse events

No patients required ICL exchange. There were a total of two cases in which the vault disappeared, but follow-up observations were made considering a shallower ACD (2.8 mm and 2.83 mm). In contrast, the highest vault was 1150 μm, and given that the patient's anterior chamber was 3.35 mm, the remaining central ACD was 1.95 mm, and all anterior chamber angles were open, ICL replacement was not performed.

## Discussion

The worldwide demand for ICL implantation is booming with the increase in myopia. The safety of this operation has always been the focus of surgeons and researchers, and it is often related to the vault. In a previous study, we proposed a vault prediction method based on the sulcus-to-sulcus diameter and lens thickness [22]. In this study, on the other hand, we took one year to validate this prediction method in four periods. This study is the one with the longest validation time span and the largest sample size among similar studies thus far.

The current study demonstrated that our prediction model yielded an overall 93.08% achievement rate for normal vault (200 to 800 μm). Moreover, the proportion of normal vault was 90.50%, 94.50%, 93.05%, and 94.55% in the four phases of the study, which demonstrated the excellent accuracy and stability of this prediction method. According to a meta-analysis by Packer, ICL size selection by WTW and ACD achieved an optimal vault ratio of approximately 83.6% [24]. A recent study by Manito et al. [25] also showed that 77.23% of cases achieved optimal vault during a 5-year period using conventional ICL size selection methods. Obviously, the results of this study are comparable with or superior to the traditional WTW-based ICL size selection method.

Over the last few years, many studies have tried to achieve an appropriate vault by exploring prediction models. Igarashi et al. [19] demonstrated that angle-to-angle (ATA) was a reliable parameter through their study of anterior segment OCT and proposed the KS formula on this basis. However, even the later verification study only shows the difference between the actual vault and the vault predicted by the KS formula. It is not clear how many cases can achieve the optimal vault using the KS formula, which is exactly what we aim to determine [26]. The NK formula was proposed by Nakamura et al. [17] based on anterior chamber width (ACW) and crystalline lens rise (CLR). The formula yielded 71% moderate vaults and 23% high-achieved vaults. Fortunately, after modification, the NK formula V2 yielded a larger percentage of moderate vaults (91.2%) [21]. It is worth noting that the sample size of the present study is much larger than that of the above study (925 eyes vs. 44 eyes and 68 eyes), and the results of this study are comparable to those of the above study. In view of the need to exclude ciliary cysts and other lesions, UBM is a necessary examination before ICL implantation. Therefore, the method of the present study could reduce the burden of patients without additional ASOCT and might be more easily accepted in clinical practice.

In addition, artificial intelligence has been used to try to perform ICL size selection in recent years. Shen et al. [27] trained and validated several machine learning models by a 3536-patient database and found that the random forest model had the highest fitting degree (*R*^2^ = 0.315) and the best prediction accuracy (82.8%); obviously, this result was not entirely satisfactory. A similar situation also appeared in the research by Kamiya et al. [28]. Moreover, similar studies are only retrospective at present. The greatest value of these studies is to confirm that the random forest model is the most effective machine learning model, but further validation is needed before clinical application.

Considering that the ICL is placed in the posterior chamber, the morphology of the ciliary sulcus and iris may affect the vault after ICL implantation. Therefore, this study summarized the occurrence frequency of four UBM features in cases with large prediction errors. ICA refers to the angle between the posterior surface of the iris and the anterior surface of the ciliary body [29]. A study by Sugiura et al. [30] reported an average ICA value of 66.3° in the normal population. Chen et al. [29] studied the UBM results of patients undergoing ICL implantation and found that the mean ICA value was 48.23° ± 16.15° in the normal vault group and 26.18° ± 16.32° in the high vault group. Further analysis showed that for every 1° decrease in ICA, the probability of vault greater than 1000 μm increased by 4%. In this study, 72.22% of eyes in the overestimate group had an ICA greater than 90°, while this proportion was only 3.7% in the underestimate group. We suspect that this might have been due to the larger ICA causing the haptics of the ICL to "sink" without being fixed in the ciliary sulcus, resulting in a reduction in vault, but this speculation needs further verification.

The effect of iris morphology on vault has rarely been reported. Iris concavity or iris backwards-bowing is often associated with pigment dispersion syndrome (PDS), and it is an important clinical sign of PDS [31]. Other studies have shown that iris concavity can also be found in healthy people [32, 33]. In this study, the incidence of iris concavity was significantly higher in the overestimate group than in the underestimate group (19.44% vs*.* 0%). We suspect that the backwards-bowing shape of the iris may increase the downwards pressure on the ICL, resulting in a lower vault. However, the cases of iris concavity in this study were not sufficient (7 eyes) and deserve further study.

An anteriorly positioned ciliary body was first proposed by Sakata et al. [34], which refers to a long ciliary process with no ciliary sulcus (ICA = 0°) as exhibiting at least two quadrants of the ciliary body. Chen et al. [29] demonstrated that an anteriorly positioned ciliary body was strongly correlated with excessive vault after ICL implantation. They explained this phenomenon by the fact that the anterior ciliary body resulted in an actually smaller sulcus-to-sulcus diameter and overcrowded posterior chamber segment [29]. This result is highly consistent with the present study.

The effect of ciliary body cysts on the vault of ICL implantation is still controversial. Zeng and his colleagues found that ciliary body cysts may lead to high vault by analysing ICL exchange cases due to excessive vault [35]. However, a study by Li et al. [36] reported that there was no statistical significance between the groups with and without cysts. The present study showed no significant differences between the two groups, but this might also be due to the small sample size (6 eyes), which requires further research.

In addition, according to our prediction formula, the vault would change by 329 μm or 394 μm for each step of ICL size. This gives us some room for adjustment in terms of ICL size selection. For example, a patient may choose a 12.6 mm ICL with a predicted vault of 680 μm; however, if he or she has an anteriorly positioned ciliary body, we tend to choose a 12.1 mm size with a predicted vault of 351 μm. Therefore, the proportions of normal vault in later seasons were slightly higher than those in the first season as our experience improved.

There are several limitations in our study. First, UBM is an examination that requires great experience and skills, and there may be some errors between different operators. The operators in this study had experience in more than 20,000 cases, and inexperience may lead to biased results. Second, all subjects in this study were Han Chinese. There are differences in ciliary body morphology between races [37]. This may lead to differences in the occurrence probability of UBM features that affect arch vault, leading to a deviation of results, which needs further verification. Third, since the focus of this study was verification of the formula, we did not carry out further analysis on UBM features that may affect the vault; this will be the subject of our next work. Finally, all ICLs in this study were placed horizontally, and different placement positions would affect the vault [38].

## Conclusions

In conclusion, this study demonstrated the accuracy and stability of our prediction formula through the validation of a large sample size and a long time span. Wide ICA, iris concavity and anteriorly positioned ciliary body may have an effect on vault.

## Data Availability

The data used in this study can be requested by contacting the corresponding author.
